# Patient participation in end-stage kidney disease care: variation over time and effects of staff-directed interventions - a quasi-experimental study

**DOI:** 10.1186/s12882-023-03313-z

**Published:** 2023-09-11

**Authors:** Caroline Hurtig, Marcus Bendtsen, Liselott Årestedt, Fredrik Uhlin, Ann Catrine Eldh

**Affiliations:** 1 Department of Health, Medicine and Caring Sciences, Linköping University, 581 83 Linköping, Sweden; 2 Department of Health and Caring Sciences, Linnaeus University, 391 82 Kalmar, Sweden; 3 Department of Nephrology, Region Östergötland, 581 85 Linköping, Sweden; 4 Department of Health Technologies, Tallinn University of Technology (TalTech), 19086 Tallinn, Estonia; 5 Department of Public Health and Caring Sciences, Uppsala University, 751 22 Uppsala, Sweden

**Keywords:** Chronic kidney failure, End-stage kidney disease, Patient participation, Patient-centred care, Patient preference, Person-centred care, Quality of health care

## Abstract

**Background:**

Among those elements establishing decent quality of care from a patient perspective, opportunities to participate in accord with one’s individual needs and preferences are central. To date, little is known the extent of preference-based patient participation in kidney care, and what facilitates optimal conditions. This study investigated i) preference-based patient participation in kidney care over time, and ii) the effects of interventions designed to enhance person-centred patient participation.

**Methods:**

A quasi-experimental study was conducted across nine kidney care sites in southeast Sweden. A cohort of 358 patients with stage IV chronic kidney disease (eGRF 15–19 ml/min) or V (eGRF < 15 mL/min) entered the study. Of these, 245 patients (with kidney replacement therapy or intermittent outpatient visits only) completed a survey on patient participation at four time points: every six months from August 2019 to May 2021, patients reported their preferences for and experiences of participation using the validated *Patient Preferences for Patient Participation* tool, the 4Ps. Between the first and second data collection points, interventions were provided for designated staff to facilitate person-centred participation, using two strategies for two subgroups at three sites each: the managers receiving a bundle of information via e-mail on patient participation in a standard dissemination procedure (three sites), or an additional half-year support program for implementation offered to 1–2 staff per site (three sites), with no intervention for a control group (three sites). The differences in 4Ps data between groups were analysed using multilevel ordinal regression.

**Results:**

Over time and across all sites, most patients’ experiences of participation fully or almost fully matched their engagement preferences (57%–90%). Still, up to 12% of patient reports indicated that their preferences and experiences were insufficiently matched: in these cases, the patients had preferred to be more involved than they had experienced, for example, in making healthcare plans and setting health-related goals. The interventions did not affect the levels of preference-based participation, but patients in the control group sites had slightly more consistent matches.

**Conclusions:**

Living with kidney failure necessitates patient engagement, but opportunities to participate in accordance with one’s preferences are not fully provided for all patients. Additional efforts to support a common understanding and to ensure person-centred patient participation is still needed.

**Supplementary Information:**

The online version contains supplementary material available at 10.1186/s12882-023-03313-z.

## Background

Patient participation is essential for establishing a decent quality of care [1–3] but is conditional on patients engaging in accordance with their preferences [4]; that is, supplying preference-based patient participation [5]. Optimal conditions for such participation have not yet been fully implemented [6] and it has been suggested that the diversity as to how patients and healthcare staff conceptualise ‘participation’ constitutes a barrier [7]. Previous studies have shown that the concept of patient participation includes sharing activities, knowledge, and experiences that facilitate learning, in addition to a mutual recognition of agreed-upon plans and goals, and self-management [8, 9]. Still, health professionals tend to focus on patient participation as primarily related to taking part in healthcare decisions and activities [10, 11]. While patients may appreciate such opportunities, they also favour participation in terms of the recognition of shared information, such as notifying staff about their experiences, learning how to deal with symptoms, and enacting such knowledge in everyday self-care [7, 12, 13]. Without a shared understanding, there is substantial risk that opportunities for patients to engage in accordance with their preferences will be missed [14, 15].

End-stage kidney disease (ESKD) is a condition with severe symptoms affecting health-related quality of life [16], which often implies regular and longstanding contact with healthcare services. ESKD therefore requires patient participation in terms of self-care [11, 17] and in the management of treatments, such as medication [18] and/or kidney replacement therapy, KRT [10, 19]. While ESKD patients can appreciate participating in decisions about their preferred type of KRT [20], there is a need for a broader perspective on patient participation in kidney care that recognises both the voice and choice of patients.

To date, few studies have addressed the conditions for preference-based patient participation in pre-dialysis and dialysis care [21]. Rather, additional efforts to facilitate a shared understanding of patient participation are suggested. The aim of this study was to investigate preference-based patient participation in ESKD care over an 18-month period, including the effects of two interventions to enhance person-centred participation directed toward designated staff enacting a facilitator role within their teams. The research questions were:

1. To what extent do patient reports represent preference-based participation over time?
2. Do interventions addressing staff to facilitate preference-based patient participation in kidney care impact the general match between patients’ preferences for and experiences of participation?

## Methods

### Study design

This quasi-experimental study represents a cohort of patients with ESKD across nine kidney care units in southeast Sweden (coded site A through I for confidentiality). The study is conducted in accord with the STROBE guidelines for reporting cohort studies (Additional file 1) [22].

### Setting and sample

The kidney sites in this study were located across county, regional, and university hospitals. Like other Swedish kidney care services, the sites were staffed primarily with registered nurses and licensed practical nurses, but the teams of healthcare professionals also included physicians, and had assigned physiotherapists, counsellors, and dietitians. The sites were all publicly funded and provided outpatient care for patients with kidney failure. The kidney replacement therapy (KRT) included both hospital-based and home-based haemodialysis, as well as peritoneal dialysis. Patients in either KRT or intermittent pre-dialysis visits were recruited from August 2019 to December 2019. Inclusion criteria were: ≤ 18 years old, stage IV chronic kidney disease (eGRF 15–19 ml/min) or V (eGRF < 15 mL/min) [23], and an ability to communicate in Swedish. The sole exclusion criterion was a cognitive impairment known to the staff (since cognitive impairment may have a negative effect on the ability to exercise and/or reflect on one’s participation as a patient).

### Recruitment procedures

During the study enrolment, a contact person at each site (a first-line manager or an assigned nurse) identified potential participants by considering the above criteria. In six units, contact details were then dispatched to the first author, who sent each candidate a letter with information about the study by post (along with a pre-paid reply envelope). Two reminder letters were sent to non-responders over a three- to four-week interval. At their preference, in three units, the contact person at the kidney site provided each candidate with the same letter of information and gave all patients general verbal reminders.

For all patients, the consent slip was sent to the first author; and no information about who had agreed to participate in the study was provided to the kidney sites. While the contact persons were asked to keep a record of how many patients received the information package, a full account of how many were approached was not reported. For reasons of confidentiality, once informed consent had been obtained, all further contact was with the first author.

### The study interventions

Prior to commencement of the study, the nine sites were allocated to one of three groups: a control group, CG (three sites); a standard dissemination group, SDG, (three sites); and a facilitated implementation group, FIG (three sites). Sites were allocated depending on the type and size of the hospital, and whether they had participated in a pilot study [7, 24]. Consequently, those sites who had been part of the pilot were positioned across the three groups. The first author, who collected data for this study, was blinded to this allocation.

- The CG received no intervention.
- The SDG received a package in October 2019, including a copy of the 4Ps tool, to facilitate a shared understanding of the patients’ preferences for, and experiences of, participation: *the Patient Preferences for Patient Participation tool* [25]. This package was sent via email (by the last author) to each site’s first-line manager and unit head. Included with this tool was an information leaflet providing a scientific background to the 4Ps and instructions on how to facilitate its usage in clinical practice. Encouraging further local work to augment patient participation as a quality-of-care element, a PowerPoint presentation that comprised both contemporary information about patient participation, and a guide to implementing the 4Ps to facilitate preference-based participation, was attached.
- The FIG were provided with the same intervention as SDG (above) plus support in facilitating preference-based participation: the managers of these three sites were asked to assign two of their local staff to act as facilitators [26]. Five internal facilitators (IFs) joined the intervention programme: two sites had two IFs each, and the third appointed one IF. These five staff (all registered nurses) were offered a half a year support programme, including a lunch-to-lunch seminar, and monthly individual or group sessions via video conference.

The FIG support programme started in October 2019 and ended in March 2020. It assembled the *Promoting Action on Research Implementation in Health Services* framework [26] and comprised seminars for the IFs on contemporary knowledge about patient participation, the 4Ps tool as a clinical tool, and a way of facilitating the implementation of evidence and policies, in accordance with national and global enterprises [27, 28]. Aiming for further dissemination of preference-based participation strategies, it encouraged the IFs to employ their previous knowledge and experience of quality improvement, entertaining a plan for implementing a more person-centred approach to patient participation, with candid sessions addressing how to identify and bridge barriers to change in clinical practice. The first two sessions comprised all the above elements, and the monthly sessions responded to what the IFs did and what they needed to facilitate implementation. The intervention was delivered by two of the members of the research team; an implementation and concept expert in patient participation, and an expert in kidney care.

The support offered to the SDG and FIG sites was framed to bridge potential gaps between current practice and optimal conditions for person-centred patient participation [29]. In addition, the FIG strategy was incorporated in the study to test whether local support (by means of IFs being trained) would trump dissemination of a support package for managers.

### Outcomes and measures

Patients were invited to report their preferences for and experiences of patient participation by means of the 4Ps tool. The 4Ps tool was developed based on conceptual, semantic, and legal aspects (including patients’ experiences) [25] and validated in ESKD care prior to this study [7]. An early evaluation of the psychometric properties of the 4Ps indicated reasonable levels of validity and reliability [8], while the version used in this study had been revised to meet higher standards (including clarification of attributes and a wider spread of response alternatives). The tool is reinforced by international standards and was considered acceptable and appropriate in the kidney care context [7, 30]. The 4Ps tool has 12 items on patient participation, reiterated in two sections (one for preferences, and one for experiences, respectively), representing an individual's situation and resources, and the healthcare encounters the individual has had, respectively [31]. Each section is accompanied by four fixed response alternatives and patients are instructed to use these to report their preference for and experience of each particular item for participation [25]. In this study, the patients were asked to consider their current healthcare contact with kidney care when completing the 4Ps.

**Fig. 1:**
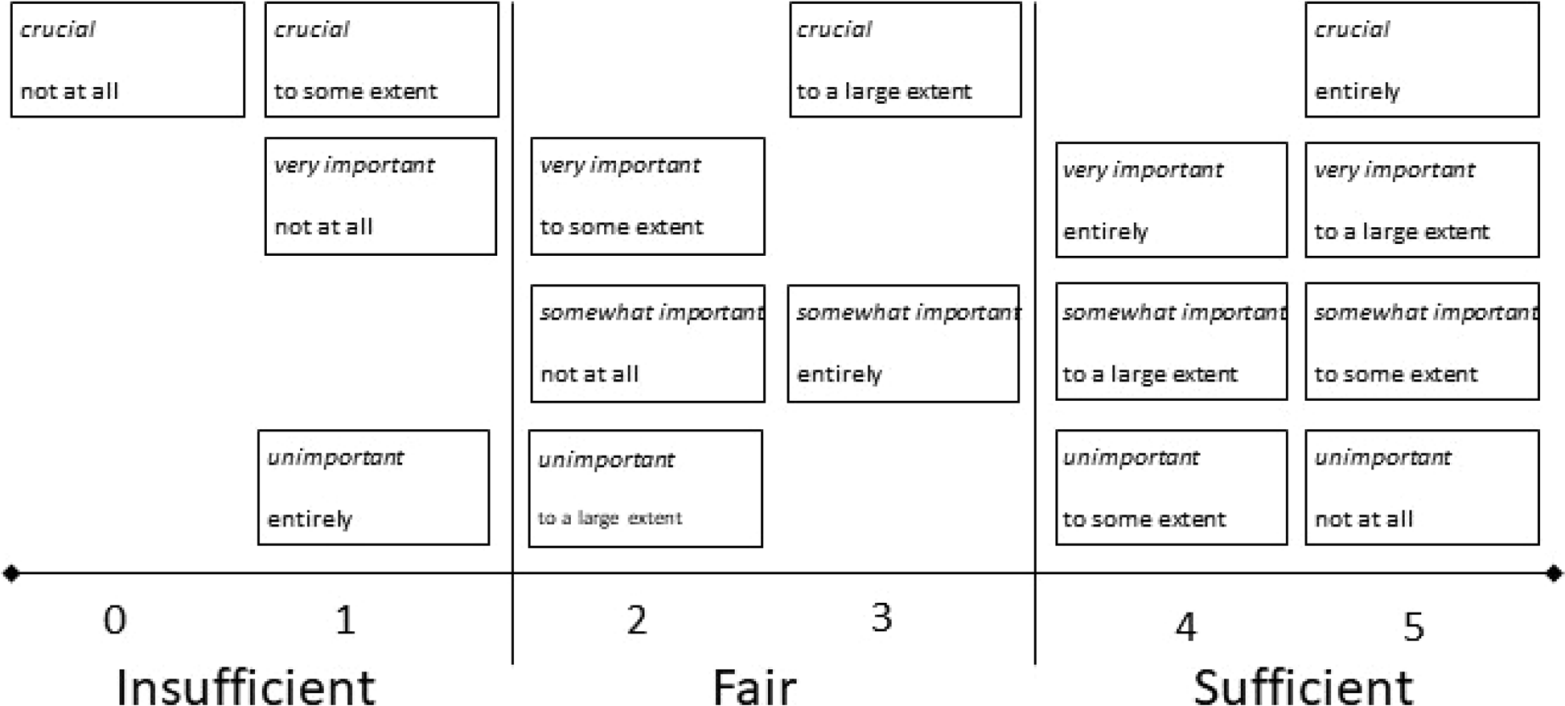
Levels and rankings for the 16 possible combinations (matches and mismatches) between patient preferences (italicised text) and patient experiences of participation (roman text). Originally published by Eldh et al. (2020) [31]

The 4Ps provides measures of preference-based patient participation, representing the degree of match or mismatch between a person’s preferences for and experiences of participation [31]: the preference-based participation ranks (0–5) are illustrated per item, deemed ‘insufficient’, ‘fair’, or ‘sufficient’ conditions for such participation (Fig. 1).

In addition to the 4Ps, demographic data was collected at baseline by means of a structured, study-specific survey with fixed response alternatives: participants reported their age, sex, years of contact with kidney care for ESKD, and years with KRT (if in dialysis).

### Data collection

Consenting patients at baseline were sent the 4Ps tool via regular postal services at four time points: August–October 2019 (that is, baseline), April 2020, October 2020, and April 2021. To ensure confidentiality, the surveys were coded and sent back in concealed envelopes to the first author via regular mail. There were no accounts of patients lacking housing stability, but altered residential addresses was likely settled by the national postal forward service (or may account for participants lost over the 18-month study duration). Two reminder letters were sent to non-responders over a three- to four-week interval, and patients were instructed to send back an empty envelope to indicate that they were not willing to participate if they preferred not to receive reminders. Data were registered in the software IBM Statistical Package for Social Sciences (SPSS) version 28, R version 4.0.4, and Stan 2.30.1 (CmdStan) prior to analyses.

### Statistical analysis

Participants were analysed in the groups to which their site was allocated (intention-to-treat). Analyses were conducted using available data, with no imputation of missing data. The Chi^2^-test, Fisher’s exact test, and the Kruskal–Wallis’s test were used to detect differences in background characteristics between CG, SDG, and FIG. A p-value of less than 0.05 was used to indicate statistical significance.

Multilevel multiple ordinal regression was used to model rankings of each of the 12 items of the 4Ps, with an interaction between follow-up interval and group allocation. The model was adjusted for age, sex, dialysis stage, and ranking at baseline, and included adaptive intercepts for unit and patient. The model was parameterised to estimate the ORs of higher rankings associated with group allocation.

Bayesian inference was used to estimate the parameters of the regression model [32]. We used Student-t priors for the covariate coefficients, centred at 0 with 3 degrees of freedom and a scale of 2.5. Adaptive intercepts were given Normal priors with mean 0 and a half-Student-t hyper-prior for the standard deviation with 3 degrees of freedom and a scale of 2.5. Medians of the marginal posterior distributions over ORs are presented, together with 2.5% and 97.5% percentiles, representing a 95% compatibility interval. We also report the posterior probability that an OR is greater or less than 1; i.e., the probability that there was a difference between groups.

## Results

### Patient characteristics

A total of 358 patients agreed to partake in the study: a majority of the patients (*n* = 185, 52%) received KRT, which encompassed regular outpatient dialysis sessions three to four times each week. The remaining 173 patients (48%) visited their outpatient care clinic intermittently, with varying number of appointments throughout a year. Informed consent was obtained from all participants, and the 4Ps was completed by each individual at least once during the four survey points. Of these participants, 351 had completed the 4Ps tool at baseline, 78 (22%) of whom were patients at the CG; 86 (24%) at the SDG; and 194 (54%) at the FIG sites. In summary, 245 patients completed the 4Ps at all four time points.

The participants were 28–94 years old (mean age of 70.5 years). Of these, 221 were men, which constituted 62% of the sample. A majority had long-term experience with kidney care (more than 3 years). Almost half the sample had intermittent outpatient care only (173, 48.5%), while a slight majority received KRT (176, 54.5%). There were 39 missing cases that did not report treatment. No statistically significant differences were found at baseline among the groups in terms of demographic variables or in rankings and levels of preference-based participation. All demographic details are presented in Table 1.

**Table 1:** Demographics of study participants

**Variable**	**Groups in the intervention** n (%)						
	**CG** 78 (22)		**SDG** 86 (24)		**FIG 194** (54)		
	**n (%),**	**mean (SD) and range**	**n (%)**	**mean (SD) and range**	**n (%)**	**mean (SD) and range**	***p*-value**
**Sex and age** n (%)							
Men 221 (62)	51 (23),	69 (13.4), 28–86	47 (21),	71 (13.2), 29–89	123 (56),	71 (12.3), 31–92	0.687
Women 134 (37)	27 (20.5)	75 (10), 57–94	38 (28),	70 (12,5), 39–89	69 (51.5)	69 (11.1), 43–89	0.064
*Missing value 3 (1)*			*1 (0.5)*		*2 (0.5)*		
**Years in kidney care,** n (%) 315 (88)							
≤ 2 years, 28 (8)	9 (3)		4 (1.5)		15 (5)		0.664
3–5 years, 106 (30)	22 (7)		24 (8)		60 (19.5)		
≥ 6 years,175 (49)	36 (11.5)		47 (15)		92 (29.5)		
*Missing value 43 (12)*	*11*		*11*		*27*		
**Years on dialysis,** n (%) 167 (90)							
≥ 2 years, 71 (43)	14 (8.5)		14 (8.5)		43 (26)		0.162
3–5 years, 61 (37)	7 (4)		22 (13.5)		32 (19)		
≥ 6 years, 34 (20)	9 (5.5)		10 (6)		15 (9)		
*Missing value, 18 (10)*	*4*		*6*		*9*		
**Treatment group,** n (%)							
Out of patient care, 173 (48)	44 (25)		34 (20)		95 (55)		0.380
KRT, 185 (52)	34 (18)		52 (28)		99 (54)		
**Type of dialysis treatment**, 146							0.459
Haemodialysis, 126 (68)	23 (16)		38 (26)		65 (45)		
Peritoneal dialysis, 19 (10.5)	3 (2)		6 (4)		10 (7)		
Transplanted, 1 (0.05)	0		0		1 (0.5)		
*Missing value, 39 (21)*	*8*		*8*		*23*		
**Distribution across the sites**	A) 36 (10); B) 68 (19); C) 90 (25); D) 30 (8.5); E) 36 (10); F) 1 (0.5); G) 19 (5); H) 29 (8); i) 49 (14)						

Note: Data expressed as n = frequencies, % = percentages, mean (SD) ± range. *CG* Control group, *SDG* Standard dissemination group, *FIG* Facilitated implementation group. *p–*value refers to Kruskal–Wallis’s test (*p* < 0.05)

### Preference-based patient participation—timeline perspectives

In Table 2, the distribution in percentages of levels and rankings of preference-based patient participation are presented for each of the four measurement points. An absolute match between the patients’ preferences and experiences of participation (rank 5, sufficient level) occurred across all four time points. In most cases, rank 5 signified that the patients considered an item in the 4Ps to be very important for participation and that they had experienced conditions for being involved in such a way to a large extent. More than 57% (*n* = 201) experienced conditions for sufficient preference-based participation (when considering both ranks 4 and 5) throughout the four time-points. The highest frequencies for preference-based participation were identified in the items ‘managing treatment’ (no. 11, n 235, 90%) and ‘performing self-care’ (no. 12, n 218, 83.8%).

**Table 2:** Distribution in percentages (%) in levels and rankings of preference-based patient participation, at the baseline and different time points after the intervention

Item (in order of the 4Ps tool)	Insufficient		Fair		Sufficient		n
	**0**	**1**	**2**	**3**	**4**	**5**	
**No. 1—Being listened to**							
Baseline	0.6	3.1	8.0	16.3	23.7	48.3	350
6 months	0.3	4.5	5.4	13.7	25.2	50.8	313
12 months	0.0	4.3	5.4	15.1	25.8	49.5	279
18 months	0.8	1.9	8.0	11.8	30.9	46.6	262
**No. 2—Experiences recognised**							
Baseline	0.9	3.4	17.2	10.3	22.4	45.7	348
6 months	0.3	3.2	16.9	8.1	25.6	45.8	308
12 months	0.4	3.2	16.4	7.8	23.5	48.8	281
18 months	0.8	0.8	10.8	10.0	24.3	53.3	259
**No. 3—Reciprocal communication**							
Baseline	0.3	6.4	8.1	15.6	23.7	46.0	346
6 months	0.3	5.2	6.8	11.6	28.1	48.1	310
12 months	0.4	5.7	8.2	13.2	22.1	50.4	280
18 months	0.8	3.8	10.4	10.4	26.2	48.5	260
**No. 4—Sharing one’s symptoms**							
Baseline	0.3	4.6	6.3	16.4	22.1	50.3	348
6 months	0.6	2.3	6.8	10.6	30.2	49.5	311
12 months	0.4	4.0	8.6	14.0	22.7	50.4	278
18 months	0.4	3.9	6.9	9.3	31.7	47.9	259
**No. 5—Explanation for symptoms**							
Baseline	0.0	8.3	14.0	11.7	22.0	44.0	350
6 months	1.0	4.2	11.3	10.7	21.4	51.5	309
12 months	0.7	7.9	14.4	9.0	17.7	50.2	277
18 months	1.9	5.4	10.7	7.7	26.8	47.5	261
**No. 6—Being told what is being done**							
Baseline	1.4	6.8	10.0	13.1	24.5	44.2	351
6 months	0.3	3.8	8.0	10.8	28.7	48.4	314
12 months	0.4	4.9	9.9	12.7	25.4	46.6	283
18 months	1.9	3.4	10.7	11.8	24.4	47.7	262
**No. 7—Learning plans**							
Baseline	1.7	8.6	11.7	12.9	22.0	43.1	350
6 months	1.3	5.8	10.9	11.8	25.2	45.0	313
12 months	0.4	7.5	14.6	12.9	23.6	41.1	280
18 months	2.3	6.1	10.3	11.5	24.5	45.2	261
**No. 8—Taking part in planning**							
Baseline	2.6	8.9	18.9	10.6	17.2	41.8	349
6 months	1.0	6.1	17.4	8.0	19.6	47.9	311
12 months	1.1	7.8	16.4	9.3	17.4	48.0	281
18 months	1.2	6.2	13.5	9.6	20.8	48.8	260
**No. 9—Phrasing own goals**							
Baseline	0.6	11.0	18.8	4.3	17.9	47.4	346
6 months	0.6	4.5	14.3	4.2	22.4	53.9	308
12 months	1.1	5.1	14.1	6.1	22.0	51.6	277
18 months	1.5	3.8	16.2	6.5	21.9	50.0	260
**No. 10—Managing symptoms**							
Baseline	0.3	11.4	15.4	15.7	14.2	43.0	351
6 months	0.6	7.9	10.2	12.7	16.5	52.1	315
12 months	0.4	6.4	14.5	15.2	11.0	52.5	282
18 months	1.2	6.6	13.9	16.2	18.5	43.6	259
**No. 11—Managing treatment**							
Baseline	0.0	2.0	4.0	10.1	34.9	49.0	347
6 months	0.0	2.5	4.8	4.8	40.1	47.8	314
12 months	00	1.1	3.9	9.3	27.9	57.9	280
18 months	0.4	0.4	4.6	4.6	36.0	54.0	261
**No. 12—Managing self-care**							
Baseline	0.9	3.7	8.0	10.9	30.3	46.3	350
6 months	0.3	1.9	6.7	8.9	32.1	50.2	315
12 months	0.0	2.1	4.6	9.5	27.9	55.8	283
18 months	0.4	1.5	8.8	5.4	32.7	51.2	260

In contrast, a ‘fair’ provision of preference-based participation (that is, ranks 2–3) was prominently identified with regards to ‘managing symptoms’ (item no. 10) and ‘partaking in planning’ (no. 8). The findings suggested that the mismatch between individuals' preferences and their experiences was due to lower levels of opportunities for engagement in relation to their preferences for these specific items.

Furthermore, some ‘insufficient’ preference-based participation (rank 0, that is, a complete mismatch) occurred: learning of plans (item no. 7) and taking part in planning (no. 8) had the highest frequencies of mismatch between patients’ preferences and experiences for a maximum of 2.6% of the patients (*n* = 9). When considering both ranks 0 and 1, almost 12% (*n* = 41) reported insufficient conditions for preference-based participation in terms of managing symptoms (no. 10)*.*

### Preference-based patient participation—study intervention perspectives

**Fig. 2:**
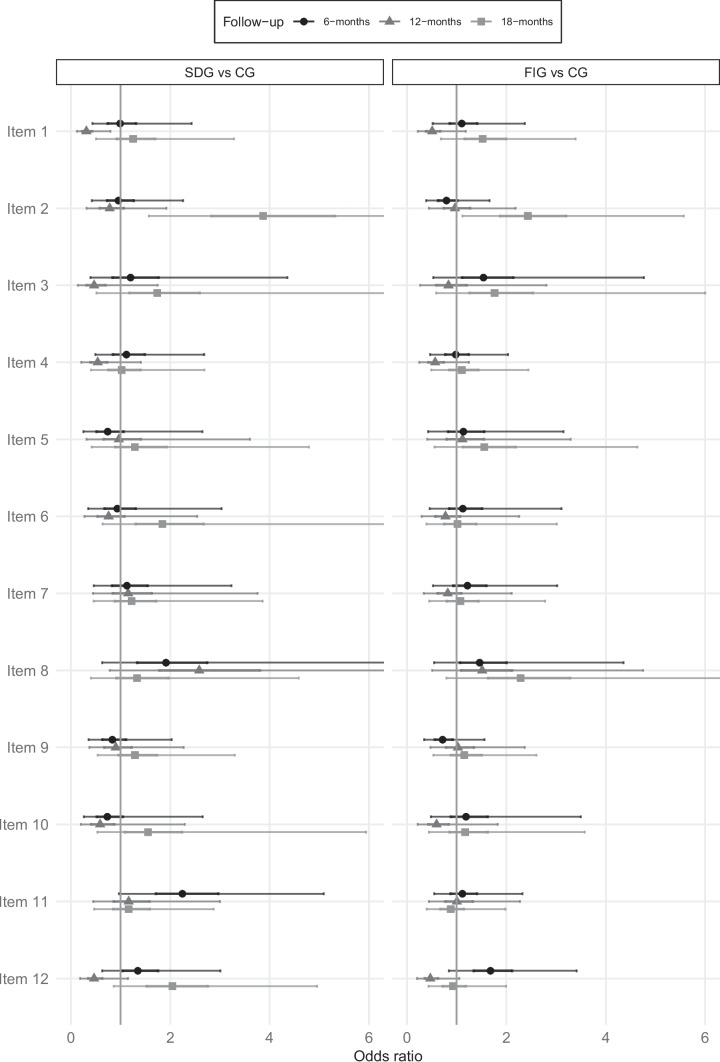
Estimated effect of the intervention on preference-based patient participation

In Table 3 and Fig. 2, the estimated effects of the interventions on preference-based patient participation are presented. Table 3 displays the contrasts between the groups at different follow-ups, generated using a model that adjusts for baseline values. This adjustment ensures that the estimates are accounted for any potential imbalances between the groups. Odds ratios (OR) are presented, comparing patients in the SDG and the FIG against patient in the CG, produced by multilevel multiple ordinal regression [31]. The OR of having a higher ranking is modelled, meaning that ORs > 1 is indicative of increased odds of higher rankings in the SDG and FIG group compared to the CG group.

**Table 3:** Estimated effect of the intervention on preference-based patient participation

	**SDG vs CG**			**FIG vs CG**		
	**6 months**	**12 months**	**18 months**	**6 months**	**12 months**	**18 months**
**No. 1—Being listened to**						
Median	0.99	0.31	1.25	1.10	0.51	1.52
95 CI	0.43; 2.43	0.12; 0.79	0.50; 3.28	0.52; 2.37	0.22; 1.18	0.68; 3.39
Post. prob	50.8%	98.9%	68.8%	60.3%	94.6%	85.7%
**No. 2—Experiences being recognised**						
Median	0.96	0.78	3.87	0.79	0.96	2.43
95 CI	0.42; 2.26	0.32; 1.92	1.57; 10.38	0.37; 1.66	0.44; 2.19	1.11; 5.57
Post. Prob	54.6%	70.8%	99.7%	73.4%	53.5%	98.6%
**No. 3—Reciprocal communication**						
Median	1.20	0.47	1.74	1.54	0.84	1.76
95 CI	0.39; 4.36	0.14; 1.75	0.51; 6.67	0.53; 4.77	0.26; 2.81	0.59; 6.00
Post. Prob	63.2%	88.8%	83.1%	81.0%	63.6%	86.7%
**No. 4—Sharing symptoms**						
Median	1.12	0.54	1.02	0.98	0.57	1.10
95 CI	0.49; 2.68	0.21; 1.41	0.41; 2.69	0.46; 2.04	0.25; 1.25	0.49; 2.45
Post. Prob	60.9%	90.8%	51.9%	52.1%	92.4%	60.0%
**No. 5—Explanations for symptoms**						
Median	0.74	0.96	1.29	1.14	1.12	1.55
95 CI	0.25; 2.65	0.31; 3.61	0.42; 4.79	0.42; 3.15	0.41; 3.29	0.55; 4.64
Post. Prob	71.3%	52.4%	67.6%	61.1%	58.5%	82.1%
**No. 6—Having explanations as to what will be/is being done**						
Median	0.93	0.76	1.84	1.12	0.77	1.02
95 CI	0.35; 3.03	0.27; 2.54	0.64; 6.51	0.46; 3.11	0.29; 2.26	0.39; 3.02
Post. Prob	55.6%	70.0%	88.1%	61.0%	70.5%	51.4%
**No. 7—Learning plans**						
Median	1.13	1.15	1.22	1.22	0.82	1.08
95 CI	0.46; 3.23	0.44; 3.76	0.46; 3.86	0.52; 3.02	0.34; 2.11	0.45; 2.78
Post. prob	60.4%	62.0%	65.9%	68.4%	68.4%	56.9%
**No. 8—Taking part in planning**						
Median	1.91	2.58	1.33	1.46	1.51	2.29
95 CI	0.63; 6.36	0.78; 8.95	0.41; 4.59	0.54; 4.36	0.51; 4.75	0.79; 6.99
Post. Prob	88.6%	94.7%	69.6%	79.2%	79.8%	94.4%
**No. 9—Phrasing own goals**						
Median	0.83	0.90	1.29	0.72	1.02	1.15
95 CI	0.36; 2.03	0.37; 2.27	0.54; 3.30	0.34; 1.56	0.47; 2.37	0.53; 2.61
Post. Prob	66.6%	58.6%	71.9%	80.8%	52.7%	64.1%
**No. 10—Managing symptoms**						
Median	0.73	0.59	1.55	1.19	0.60	1.17
95 CI	0.26; 2.65	0.20; 2.29	0.53; 5.94	0.48; 3.50	0.22; 1.83	0.44; 3.57
Post. prob	72.3%	81.3%	80.0%	65.4%	84.0%	63.4%
**No. 11—Managing treatment**						
Median	2.24	1.16	1.16	1.12	1.01	0.88
95 CI	0.97; 5.09	0.45; 3.00	0.47; 2.87	0.55; 2.32	0.44; 2.27	0.40; 1.98
Post. prob	97.2%	63.5%	62.7%	61.7%	50.5%	62.4%
**No. 12—Managing self-care**						
Median	1.35	0.47	2.04	1.68	0.47	0.92
95 CI	0.63; 3.01	0.19; 1.15	0.86; 4.96	0.84; 3.41	0.20; 1.05	0.43; 1.99
Post. prob	77.6%	95.2%	94.6%	93.7%	96.7%	58.6%

Note: Based on measures of the 4Ps tool. Median = the median of the posterior distribution; 95% CI = 95% compatibility intervals defined by the 2.5% and 97.5% percentiles of the posterior distribution; Post. prob. = the posterior probability that the OR is > / < 1 in the direction of the median

#### The standard dissemination group versus vs the control group

When comparing the patient outcomes in the SDG sites with those in CG sites (Table 3 and Fig. 2) there was evidence at 12 months after baseline indicating that those cared for in the CG sites had higher matches for preference-based patient participation than patients in the SDG sites regarding ‘being listened to’ (item no. 1), ‘having reciprocal communication’ (no. 3), ‘managing symptoms’ (no. 4) and ‘performing self-care’ (no. 12). However, these differences were not apparent after 18 months, and the patients in the SDG sites were more likely to have higher matches than the CG sites patients for item no. 12. In addition, the patients in the SDG sites had higher matches for ‘my experiences recognised’ (item no. 2) at 18 months and ‘taking part in planning’ (no. 8) at 12 months.

#### The facilitated intervention group versus the control group

When comparing patient outcomes in the FIG sites with the CG sites patients (Table 3, Fig. 2), there was some evidence that those patients cared for within the CG sites had higher matches for preference-based patient participation than those within the FIG sites when it came to: ‘being listened to’ (item no. 1); ‘managing symptoms’ (no. 4), and ‘performing self-care’ (no. 12). There were no data to explain for this, although we note that in retrospect, the local hospitals were predominantly within the CG (two sites out of three, whereas the SDG and FGI had one local hospital each). For patients in the FIG sites, on the other hand, preference-based participation (that is, a match between preferences and experiences) transpired for items ‘my experiences being recognised’ (no. 2) and ‘taking part in planning’ (no. 8) at 18 months.

## Discussion

This paper aimed to investigate preference-based patient participation in kidney care over time, and to examine the effects of staff-directed interventions designed to enhance person-centred patient participation. The key findings demonstrate that a majority of the patients had a sufficient provision of preference-based patient participation, meaning that their experiences of participation to a large extent or completely matched their preferences. However, up to 12% of patients had insufficient provision, suggesting that their experiences did not match their preferences. Notably, the staff-directed interventions did not have an impact on preference-based participation.

Globally, healthcare services are encouraged to provide patients with opportunities to partake in their own care in alignment with their own terms, including their preferences [33]. Moreover, patients are encouraged to interact with healthcare professionals and to employ strategies and activities to monitor their own health and well-being [34]. As patient participation is essential, it is promising to note that participation is more often than not facilitated in the kidney care context. Still, there remain additional prospects to facilitate preference-based participation for more patients, and to identify the barriers and enablers for such conduct [3, 35, 36].

A ‘sufficient’ provision of preference-based patient participation in kidney care was generally found in relation to attributes of participation signified by performing activities, including managing self-care as a patient. These findings align with a previous cross-sectional study [21] and confirm that important opportunities for managing self-care do exist, potentially delaying illness progression and promoting good health outcomes [37]. Patients with long-term conditions and healthcare contacts have suggested that participation is facilitated when they have the time and opportunity to share and assimilate knowledge [37] and use this in relation to strategies of managing self-care [38]. Patients with ESKD usually experience a slow disease trajectory of the disease [39], entailing regular meetings and long-term relationships with their healthcare team, which provide ample opportunities to facilitate preference-based participation in the kidney care context.

Our findings provide little evidence that any of the interventions directed toward designated staff facilitated more consistent or coherent preference-based patient participation. Similar training of staff in primary care [40] confirm that any change is a slow and challenging process. In this case, a parallel (ongoing) process evaluation with managers, staff, and the internal facilitators (IFs) (in the FIG sites), indicate that in the SDG sites no staff were involved. Still, in the FIG sites, few staff beyond the IFs became engaged in the intervention. While the IFs considered their own conduct vis-à-vis patients’ participation (enabling further preference-based participation for some but only few patients), they experienced a lack of local support. Thus, few procured a strategy facilitating more person-centred patient participation practices in general in their sites during the intervention period. A careful evaluation of how staff-directed interventions are received, adopted, and delivered is required, so as to better understand whether and how the dissemination of information and/or the support programme facilitated the IFs and their fellow staff to change in the provision of opportunities for patient participation [41].

In contrast to the high provisions of ‘sufficient’ levels of preference-based participation, our study also showed that patients’ experiences and preferences were the least likely to match in terms of taking part in planning, phrasing personal goals, and managing symptoms: the patients either had experiences that exceeded their preferences, or fewer conditions for participation than they would have preferred. It is suggested to be better to provide ample conditions for participation, rather than fewer conditions than patients may prefer [31], for example, more information and knowledge can still enhance health literacy; that is, a patient’s ability to understand and manage their illness and treatment [42]. While health literacy can improve health status and sustain improved quality of life [43], patients with ESKD have been found to lack opportunities for optimal health literacy [44]. Consequently, a healthcare context where one’s preferences for being engaged matters is not immediately evident. While one’s preferences for participation can vary [45], patients should be encouraged to participate in accord with and to the extent they are comfortable sufficed by, supported by the focus of healthcare staff on the matters most important to patients.

‘Insufficient’ levels of preference-based participation are not unique to kidney care but also occur in other care: high frequencies of mismatches have transpired between patients’ preferences and experiences managing symptoms and taking part in planning in both primary and surgical care [40, 46]. For patients with ESKD, who often suffer from fatigue [47, 48], a recognition of their command in making treatment plans is necessary. Optimal conditions for engagement and a recognition of the potential for further ways to participate may arise during repeated and long-lasting ESKD treatments, but a better understanding of how best to support common understanding is still needed.

### Methodological considerations

The nine sites partaking in this study represent 15% of dialysis care in Sweden, although only 3% of the approximately 4000 individuals undergoing KRT [49]. However, a high percentage of those signing up remained for the full study, despite the potential for dropout due to ESKD-induced or treatment-induced fatigue [47, 48]. Furthermore, participants were representative in terms of gender and age, as more men than women suffer from ESKD [49, 50] and the condition affecting older people to a greater extent than younger individuals [49]. This indicates a potential for the findings to be transferable, particularly as the 4Ps tool has been suggested to be valid for capturing preferences and experiences of participation in a broad sense [30] which corresponds to person-centred patient participation [45]. Nonetheless, the study findings are constrained when it comes to potential transferability of person-centred patient participation among patients with cognitive impairments, due to the lack of patient reports from those with such mental conditions. With limited details as for when the patients with only intermittent outpatient visits had had their last encounter, the data call for some caution: recall bias may influence one’s experience of participation, even if important events (such as, seemingly any healthcare interaction regarding one’s health if living with ESKD) incline a lasting impression [51].

Parts of this study, more specially the data collection at 12 and 18 months, was conducted during the COVID-19 pandemic. It may have affected the healthcare services that the patients encountered, with many hospitals inclined to direct any staff available to serve temporary pandemic units, and more patient contacts in kidney care offered via telephone (or digital) consultations. This may point to the potential benefits of having KRT in a control site if less transmission of staff took place within local hospitals [52].

### Implications for practice

While patients with ESKD seemed to often experience opportunities for involvement consistent with their preferences, there was variance regarding at what time point and which of the particular attributes of patient participation was satisfactory facilitated. Consequently, there is a need to further address and recognise the ways in which, and to what extent, patients can and wish to engage in their own care and treatment.

## Conclusion

Living with ESKD necessitates patient engagement, and the patient reports most often indicated a match between their preferences for and experiences of participation. Yet, some degree of mismatch did occur over time in all the attributes conceptualising patient participation. This indicates that the opportunities patients have to participate in care in the ways in which they can and will engage may be hampered. The interventions designed to enable staff to optimise patients’ conditions for involvement did not evidently affect preference-based participation in this study.

### Supplementary Information

**Additional file 1.**

## Data Availability

Data are available from the corresponding author upon reasonable request.
