# Supplementary material for: Patient participation in end-stage kidney disease care: variation over time and effects of staff-directed interventions - a quasi-experimental study
Source: BMC Nephrol. 2023 Sep 11;24:265. doi: 10.1186/s12882-023-03313-z (PMC10494352; doi:10.1186/s12882-023-03313-z)
Supplement: Supplementary file 1 — Additional file 1. [file 12882_2023_3313_MOESM1_ESM.pdf]

STROBE Statement—checklist of items that should be included in reports of observational studies.

	Item No.	Recommendation	Yes/No/NA, Page No.
**Title and abstract**	1	(*a*) Indicate the study's design with a commonly used term in the title or the abstract	Yes, Page No. 1.
		(*b*) Provide in the abstract an informative and balanced summary of what was done and what was found	Yes, Page No. 2.
**Introduction**			
Background/rationale	2	Explain the scientific background and rationale for the investigation being reported	Yes, Page No.3.
Objectives	3	State specific objectives, including any prespecified hypotheses	Yes, Page No 3 and 4.
**Methods**			
Study design	4	Present key elements of study design early in the paper	Yes, Page No.2 and 4.
Setting	5	Describe the setting, locations, and relevant dates, including periods of recruitment, exposure, follow-up, and data collection	Yes, Page No. 4
Participants	6	(*a*) *Cohort study*—Give the eligibility criteria, and the sources and methods of selection of participants. Describe methods of follow-up	Yes, Page No. 4, 5 and 6.
		(*b*) *Cohort study*—For matched studies, give matching criteria and number of exposed and unexposed	NA
Variables	7	Clearly define all outcomes, exposures, predictors, potential confounders, and effect modifiers. Give diagnostic criteria, if applicable	Yes, Page No. 6 and 7.
Data sources/ measurement	8*	For each variable of interest, give sources of data and details of methods of assessment (measurement). Describe comparability of assessment methods if there is more than one group	Yes, Page No. 5, 6 and 7
Bias	9	Describe any efforts to address potential sources of bias	Yes, Page No.7 and 16
Study size	10	Explain how the study size was arrived at	Yes, Page No. 4

Quantitative variables	11	Explain how quantitative variables were handled in the analyses. If applicable, describe which groupings were chosen and why	Yes, Page No. 7
Statistical methods	12	(*a*) Describe all statistical methods, including those used to control for confounding	Yes, Page No. 7
		(*b*) Describe any methods used to examine subgroups and interactions	Yes, Page No. 7
		(*c*) Explain how missing data were addressed	Yes, Page No. 7
		(*d*) *Cohort study*—If applicable, explain how loss to follow-up was addressed	Yes, Page No. 7
Statistical methods	12	(*a*) Describe all statistical methods, including those used to control for confounding	Yes, Page No. 7
		(*e*) Describe any sensitivity analyses	NA
Participants	13*	(a) Report numbers of individuals at each stage of study—eg numbers potentially eligible, examined for eligibility, confirmed eligible, included in the study, completing follow-up, and analysed	Yes, Page No. 8
		(b) Give reasons for non-participation at each stage	NA
		(c) Consider use of a flow diagram	
Participants	13*	(a) Report numbers of individuals at each stage of study—eg numbers potentially eligible, examined for eligibility, confirmed eligible, included in the study, completing follow-up, and analysed	Yes, Page No. 8
Descriptive data	14*	(a) Give characteristics of study participants (eg demographic, clinical, social) and information on exposures and potential confounders	Yes, Page No. 8
		(b) Indicate number of participants with missing data for each variable of interest	Yes, Page No. 8
		(c) *Cohort study*—Summarise follow-up time (eg, average and total amount)	NA
Outcome data	15*	*Cohort study*—Report numbers of outcome events or summary measures over time	Yes, Page No. 9 – 13.
Main results	16	(*a*) Give unadjusted estimates and, if applicable, confounder-adjusted estimates and their precision (eg, 95% confidence interval). Make clear which confounders were adjusted for and why they were included	Yes, Page No. 11-13.
		(*b*) Report category boundaries when continuous variables were categorized	NA
		(*c*) If relevant, consider translating estimates of relative risk into absolute risk for a meaningful time period	NA
Other analyses	17	Report other analyses done—eg analyses of subgroups and interactions, and sensitivity analyses	Yes, Page No. 11-13
Key results	18	Summarise key results with reference to study objectives	Yes, Page No. 2 and 14

Limitations	19	Discuss limitations of the study, taking into account sources of potential bias or imprecision. Discuss both direction and magnitude of any potential bias	Yes, Page No. 16
Interpretation	20	Give a cautious overall interpretation of results considering objectives, limitations, multiplicity of analyses, results from similar studies, and other relevant evidence	Yes, Page No.14-16.
Generalisability	21	Discuss the generalisability (external validity) of the study results	Yes, Page No. 16
**Other information**			
Funding	22	Give the source of funding and the role of the funders for the present study and, if applicable, for the original study on which the present article is based	Yes, Page No.17
